# Self‐reported accidental allergic reactions among patients with challenge‐verified food allergy

**DOI:** 10.1002/clt2.70067

**Published:** 2025-06-18

**Authors:** Sebastian Vigand Svendsen, Annemarie Schaeffer Senders, Athamaica Ruiz Oropeza, Annmarie Lassen, Carsten Bindslev‐Jensen, Charlotte G. Mortz

**Affiliations:** ^1^ Department of Dermatology and Allergy Centre Odense University Hospital University of Southern Denmark Odense Denmark; ^2^ Department of Emergency Medicine Odense University Hospital Odense Denmark

**Keywords:** accidental allergic reaction, allergic reactions, anaphylaxis, food allergy, oral food challenge

## Abstract

**Background:**

Food allergy affects up to 6% of the population and emergency department visits due to accidental food‐allergic reactions are increasing. This study evaluated accidental allergic reactions outside the hospital and the number of hospitalizations in food allergic patients as well as the pattern before and after the diagnosis of food allergy by oral food challenge (OFC).

**Methods:**

An electronic questionnaire concerning accidental allergic reactions was sent to 785 patients with challenge verified peanuts, hazelnuts, cow's milk and/or hen's egg allergies at the Allergy Centre, Odense University Hospital, Denmark.

**Results:**

In total, 51% (402/785) responded. Among the 357 who reported at least one accidental allergic reaction, 51.5% (184/357) reported a total of six or less reactions, whereas 22.4% (80/357) had experienced a total of ≥21 reactions. Skin symptoms were commonly reported by children/adolescents (*n* = 277), whereas symptoms from all other organ systems were more frequently reported by adults (*n* = 80). In total, 61.6% (220/357) experienced at least one accidental allergic reaction, requiring immediate medical attention, which decreased from 77.3% (170/220) before to 55% (121/220) after establishment of the food allergy diagnosis by OFC. A concomitant proportional increase in the number of hospitalizations was identified (63.5% (108/170) to 72.7% (88/121)). Limitations: We had no exact data on the timing of the accidental allergic reactions for the individual allergens.

**Conclusion:**

Accidental food‐allergic reactions are common and often severe. After the diagnostic OFC, the number of patients with reactions decreased, and the proportion of hospitalizations increased, indicating improved disease and healthcare management.

## 1 INTRODUCTION

Food allergy is a growing concern in Western countries, affecting up to 6% of children and adults in Europe.^1^, ^2^, ^3^ In childhood, cow's milk, hen's egg, peanuts, and tree nuts are among the most common foods causing food allergy,^4^, ^5^ of which peanuts and tree nut allergies often persist into adulthood.^6^

Individuals with food allergy are, despite attempts to avoid the triggering foods,^7^ at daily risk of accidental exposures,^8^ potentially causing life‐threatening anaphylaxis.^9^ Oral food challenge (OFC) is not always needed for the diagnosis of food allergy^10^ but is important for threshold determination^11^ and can help guide the patient on the appropriate level of avoidance for example, traces of allergen. According to the literature, 42%–70% of children,^12^, ^13^ 85% of adolescents,^13^ and 51% of adults^14^ with food allergy have experienced severe allergic reactions with multi‐organ symptoms. Food‐induced allergic reactions are responsible for increased contact with emergency departments,^15^ leading to a fourfold increase in food‐related hospitalizations among adolescents within the last decades.^16^ Furthermore, children^17^ and adults^18^ experience impaired quality of life due to food allergy, although sometimes due to overestimating the risk of fatal reactions.^19^ Current knowledge of accidental allergic reactions outside a hospital setting is scarce, and the relationship to diagnostic OFC is unknown.

This study aims to characterize the accidental food‐allergic reactions outside the hospital setting among patients with peanuts, hazelnuts, cow's milk and/or hen's egg allergies before and after a positive OFC as part of the diagnostic evaluation as well as the number of hospitalizations.

## 2 METHODS

### 2.1 Study population

Patients of all ages with a positive OFC to peanuts, hazelnuts, cow's milk, and/or hen's egg at the Department of Dermatology and Allergy Centre, Odense University Hospital, Denmark, from January 2001 through June 2015, were included in the study. These patients received an electronic questionnaire in October 2015, followed by two reminders within two months before the study was closed in February 2016.

### 2.2 Oral food challenge

The open OFCs were performed from 2001 through 2015, following EAACI guidelines^7^ in titrated challenge dosages (0.25, 2.5, 24.6, 49.2, 172.2, 319.8, 639.6, and 1476 mg peanuts protein, 0.13, 1.3, 13.2, 26.4, 92.4, 171.6, 343.2, 660 mg hazelnuts protein; 0.03, 0.09, 0.17, 0.34, 0.68, 1.36, 2.72, and 5.44 mg cow's milk protein; 1.25, 5.0, 28.5, 57, 114, 285, 570, and 4560 mg hen's egg protein) with a minimum of 30 min interval between administrations. Stopping criteria were confined to objective allergic signs.^11^

The food allergy diagnosis was established upon the first positive OFC for any of the four food allergens.

### 2.3 The questionnaire

The electronic questionnaire was designed to gather information about peanuts, hazelnuts, milk, and hen's egg allergies. It contained core questions about the individual's allergies, the symptoms experienced, the need for acute medical attention and potential treatments, co‐morbidity, and co‐factors during accidental allergic reactions outside hospital settings. If the individual has had more than one allergic reaction, the most severe reaction was considered for the overall analysis. The patients were asked to report a total number of accidental allergic reactions (severe and non‐severe, as specified below) and a detailed description of their first, most recent, and most severe allergic reactions that they had experienced. Therefore, it was not possible to specify the timing of individual eliciting allergens if patients were allergic to more than one food allergen. The questionnaire underwent evaluation and testing by healthcare professionals, including medical doctors and nurses, with expertise in food allergy. Additionally, it was tested by ten food‐allergic patients and their families to ensure the quality before being sent to potential study participants.

In the questionnaire, we defined a severe allergic reaction as an accidental allergic reaction requiring immediate medical attention outside or within a hospital setting, regardless of the administered treatment. The severity of self‐reported symptoms during the accidental allergic reactions was graded using Sampson's severity score.^20^ Anaphylaxis was defined as a score of 4–5, whereas scores 1–3 were categorized as mild‐moderate allergic reactions.^20^

### 2.4 Statistical analyses

The Pearson chi‐square (*χ* ^2^) test was used for data comparison, and the *χ* ^2^ trend test (Cochran–Mantel–Haenszel tests) was used for testing trends. The level of statistical significance for the two‐sided test was *p*‐value <0.05. Data analysis was performed using STATA version 17 (Stata Corporation LP®, Texas, USA).

### 2.5 Ethics

All patients were registered in the Allergy Centre Database, containing clinical OFC information, including allergens, time and dosages administered, and elicited symptoms and signs. Informed consent was obtained from all patients or parents of children. The study was approved by the Regional Committees on Health Research Ethics for Southern Denmark (S‐20120203) and the Danish Data Protection Agency (12/26172).

## 3 RESULTS

### 3.1 Demographics of included food‐allergic patients

**FIGURE 1:**
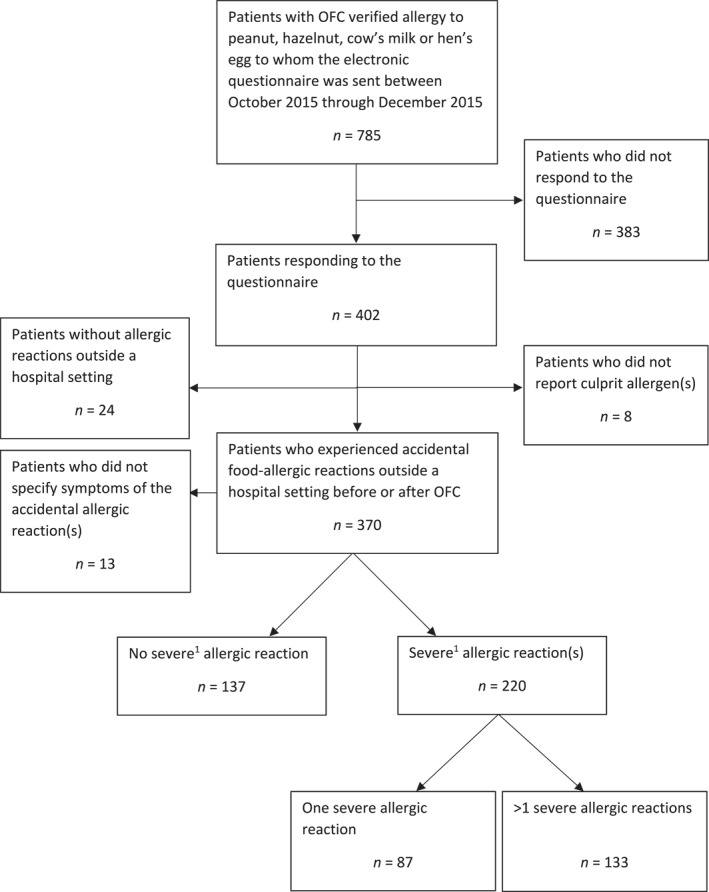
Flow diagram of patients with allergy to peanuts, hazelnuts, cow's milk, and hen's egg diagnosed by oral food challenge between 2001 and 2015 at the Department of Dermatology and Allergy Centre, Odense University Hospital, Denmark. OFC, oral food challenge. ^1^Severe allergic reactions were defined as accidental allergic reactions to peanuts, hazelnuts, cow's milk or hen's egg outside the hospital settings that required immediate medical attention.

In total, 402 patients (42.8% females) of 785 eligible answered the questionnaire, corresponding to a response rate of 51.2% (402/785); see Figure 1. Children and adolescents (≤17 years) or their parents accounted for 77.4% (311/402) of the respondents. Overall, the questionnaires were answered by patients (28.1%, 113/402), parents (50%, 200/402), patient and parent(s) in cooperation (21.9%, 88/402), or unknown respondent (1/402). There was an equal distribution between the responders and non‐responders, considering sex, age, OFC cumulative thresholds of allergens, and Sampson's severity score during the OFC with the lowest cumulative threshold (Table 1).

**TABLE 1:** Baseline characteristics of the study population at the time of the questionnaire distribution, including all patients with a positive oral food challenge to peanuts, hazelnuts, cow's milk and/or hen's eggs, and questionnaire responders.

	All patients (*n* = 785)	Questionnaire, % (*n*)		
		Non‐responders (*n* = 383)	Responders *(n* = 402)	*χ* ^2^‐test *p*‐value
Sex, *n* (%)				
Female	316	45.6 (144)	54.4 (172)	0.138
Male	469	51.0 (239)	49.0 (230)	
Age, n (%)				
≤ 17 years	598	48.0 (287)	52 (311)	0.425
≥ 18 years	187	51.3 (96)	48.7 (91)	
Lowest cumulative threshold of oral food challenge, stratified by culprit, n (%)				
Peanuts	351	48.7 (171)	51.3 (180)	
≤2311 mg	277	49.1 (136)	50.9 (141)	0.783
>2311 mg	74	47.3 (35)	52.7 (39)	
Hazelnuts	155	44.5 (69)	55.5 (86)	
≤2311 mg	107	43.0 (46)	57.0 (61)	0.568
>2311 mg	48	47.9 (23)	52.1 (25)	
Cow's milk	104	49.0 (51)	51.0 (53)	
≤18.5 mL	53	49.1 (26)	50.9 (27)	0.997
>18.5 mL	51	49.0 (25)	51.0 (26)	
Hen's egg	336	53.9 (181)	46.1 (155)	
≤1805 mg	182	52.8 (96)	47.3 (86)	0.654
>1805 mg	154	55.2 (85)	44.8 (69)	
Sampson's severity score during the oral food challenge with the lowest cumulative threshold^a^, *n* (%)				
1–3	560	49.8 (279)	50.2 (281)	0.397
4–5	146	45.9 (67)	54.1 (79)	

Abbreviations: E, Hen's egg; H, Hazelnuts; M, Cow's milk; P, Peanuts.

^a^ For *n* = 79 (42/79 responded to the questionnaire), Sampson's severity scores were not available.

At the time of the questionnaire response, 61.7% (251/394) were present or previous peanuts allergic, 52.5% (207/394) were present or previous hazelnuts allergic, whereas 41.5% (167/394) and 29.2% (115/394) were present or previous allergic to hen's egg or cow's milk, respectively. Approximately 80% reported persistent peanuts or hazelnuts allergies, whereas only 25% and 36% reported persistent cow's milk or hen's egg allergies, respectively.

Eight patients did not specify the eliciting allergen(s) of allergic reactions, of whom five patients had positive OFCs to hazelnuts, one patient had a positive OFC to peanuts and hazelnuts, one patient had a positive OFC to peanuts, and one patient had a positive OFC to hazelnuts, cow's milk, and hen's egg. Among all questionnaire responders (*n* = 402), only 6% (24/402) reported no accidental allergic exposures to the culprit allergen(s), whereas 94% (378/402) had experienced ≥1 accidental allergic reaction. However, 5.2% (21/402) provided incomplete responses (including the *n* = 8 patients who did not report the culprit(s)) of whom and *n* = 13 did not specify the allergic symptoms, including eight patients who were allergic to one allergen; three were allergic to peanuts, two were allergic to eggs, two were allergic to hazelnuts, and one was allergic to milk. Additionally, five of the patients were concomitantly allergic to multiple allergens: one patient was allergic to egg, milk, and peanuts; one was allergic to egg and milk; one was allergic to egg and peanuts; one was allergic to egg and hazelnuts; and one was allergic to peanuts and hazelnuts. Thus, the study population consisted of 357 patients (92%, *n* = 277 aged ≤17); Figure 1.

### 3.2 Characterization of the accidental allergic reactions

**FIGURE 2:**
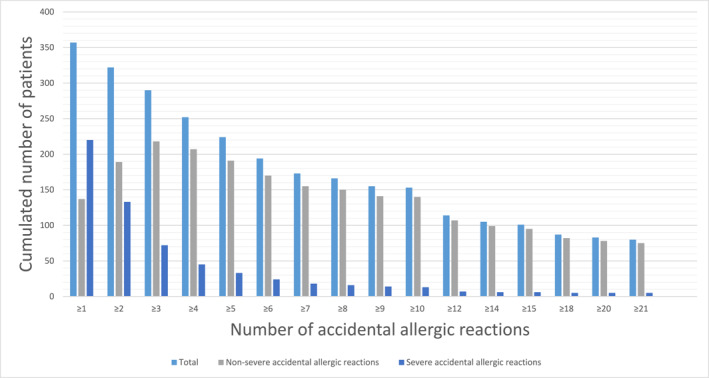
The cumulative number of challenge‐verified allergic patients (*n* = 357) who have experienced accidental allergic reactions outside the hospital setting to the culprit allergens peanuts, hazelnuts, cow's milk and/or hen's egg. Each column represents the cumulative number of patients (given on the *y*‐axis) who reported experiencing a total number of accidental allergic reactions corresponding to or higher than the given number on the *x*‐axis. Severe allergic reactions were defined as accidental reactions to any peanuts, hazelnuts, cow's milk, or hen's egg outside the hospital setting requiring immediate medical attention (e.g., attendance to the acute ward). Among all the patients with at least one accidental allergic reaction outside a hospital setting, 137 (38.4%) had not experienced a severe reaction requiring immediate medical attention to peanuts, hazelnuts, cow's milk, or hen's egg. Patients with 1–2 severe allergic reactions were subsequently presented in the non‐severe column if the number of non‐severe accidental allergic reactions was higher than that of severe reactions.

All included respondents had experienced at least one accidental allergic reaction (Figure 2) of any severity, predominantly 1–6 reactions (184/357, 51.5%); however, 22.4% (80/357) of patients reported having experienced at least 21 reactions. In total, 61.6% (220/357) of patients had experienced at least one accidental allergic reaction that required immediate medical attention, predominantly 1–3 reactions (175/220, 79.5%); Figure 2. We found no association between the experience of accidental food‐induced anaphylaxis and sex or the lowest cumulative OFC thresholds (stratified as in Table 1); data not shown.

**FIGURE 3:**
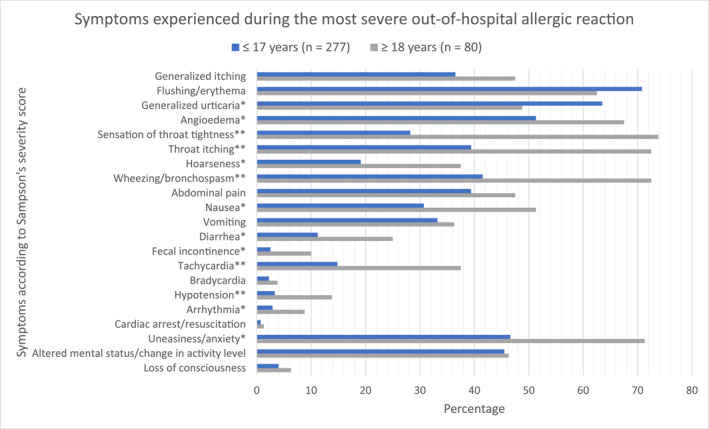
Symptoms listed by organ system comparing children and adolescents (≤17 years) versus adults (≥18 years) among all patients (*n* = 357) who specified the symptomatology experienced during the accidental allergic reactions to peanuts, hazelnuts, cow's milk or hen's egg outside a hospital setting. If the patient experienced symptoms during multiple accidental allergic reactions to peanuts, hazelnuts, cow's milk, or hen's eggs, the reaction with the highest Sampson's severity score was presented. *χ ^2^‐test *p*‐value <0.05, comparing ≤17 years versus ≥ 18 years. **χ ^2^‐test *p*‐value <0.001, comparing ≤17 years versus ≥ 18 years.

The reported symptoms during the accidental allergic reactions outside a hospital setting with the highest Sampson's score were compared between children/adolescents (≤17 years) and adults (≥18 years); Figure 3. Respiratory (sensation of throat tightness, throat itch, and bronchospasm) and cutaneous (flushing, urticaria, angioedema, skin itching) symptoms as well as uneasiness/anxiety were among the most commonly reported symptoms during the most severe accidental allergic reactions occurring outside a hospital setting. Generalized urticaria was the only symptom more frequently reported among children/adolescents compared to adults (63.5% vs. 48.8%, respectively, *p* = 0.017). On the contrary, adults reported symptoms from all other organ systems more frequently (*p* < 0.05).

### 3.3 Accidental allergic reaction(s) and hospitalization(s) before and after oral food challenge

**FIGURE 4:**
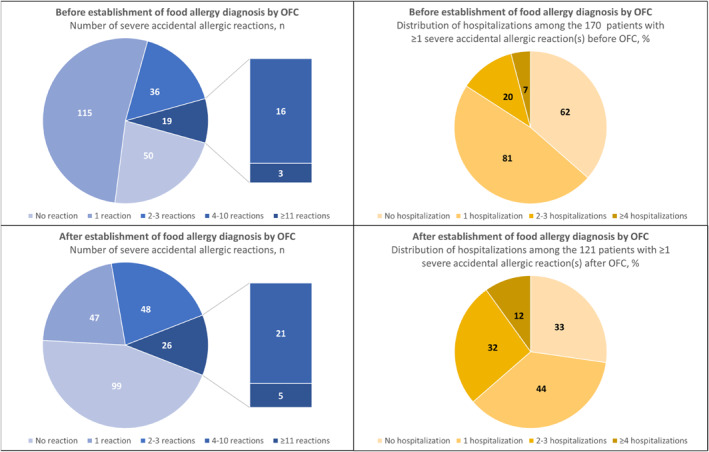
Distributions of all patients (*n* = 220) with at least one accidental allergic reaction to any of the four culprit allergens outside a hospital settings, requiring immediate medical attention, before and after diagnosis of allergy to peanuts, hazelnuts, cow's milk, and hen's egg by oral food challenge.

Figure 4 presents the number of patients with severe accidental allergic reactions and hospitalizations before and/or after the establishment of the food allergy diagnosis by OFC (the first positive challenge to any of the four food allergens). Among the 220 patients with at least one accidental allergic reaction requiring immediate medical attention (Table 2), the proportion decreased from 77.3% (170/220) to 55% (121/220) before and after diagnostic OFC, respectively. However, the proportion of patients reporting ≥2 severe accidental allergic reactions increased from 25% (55/220) to 33.6% (74/220) in the same period. The overall proportion of hospitalization(s) due to severe allergic reactions increased from 63.5% (108/170) before to 72.7% (88/121) after the establishment of the food allergy diagnosis by OFC to the culprit allergen.

**TABLE 2:** Severity of the accidental allergic reactions and symptoms before and after establishment of food allergy diagnosis by OFC to peanuts, hazelnuts, cow's milk or hen's egg in the *n* = 93 patients with an allergic reaction both before and after the OFC.

Symptoms that occurred during allergic reactions outside a hospital setting before and after the first positive OFC to the culprit allergen			
	Before OFC % (*n*)	After OFC % (*n*)	*χ*2‐test *p*‐value^a^
Sampson's severity score of the accidental allergic reactions^b^			
1–3	28 (26)	21.5 (20)	0.308^c^
4–5	72 (67)	78.5 (73)	
Skin, subcutaneous tissue and mucosa			
Generalized itching	43 (40)	30.1 (28)	0.068
Flushing/erythema	79.6 (74)	47.3 (44)	<0.001
Generalized urticaria	72 (67)	40.9 (38)	<0.001
Angioedema	71 (66)	45.2 (42)	<0.001
Respiratory tract			
Sensation of throat tightness	41.9 (39)	43 (40)	0.882
Throat itching	55.9 (52)	45.2 (42)	0.142
Hoarseness	26.9 (25)	31.2 (29)	0.518
Wheezing/bronchospasm	17.2 (16)	43 (40)	<0.001
Gastrointestinal tract			
Abdominal pain	44.1 (41)	36.6 (34)	0.295
Nausea	33.3 (31)	32.3 (30)	0.876
Vomiting	15.1 (14)	25.7 (23)	0.098
Diarrhea	16.1 (15)	7.5 (7)	0.069
Fecal incontinence	7.5 (7)	3.2 (3)	0.193
Cardiovascular system			
Tachycardia	21.5 (20)	21.5 (20)	1
Bradycardia	2.2 (2)	2.2 (2)	1
Hypotension	2.2 (2)	5.4 (5)	0.248
Arrhythmia	2.2 (2)	2.2 (2)	1
Cardiac arrest/resuscitation	1.1 (1)	2.2 (2)	0.561
Central nervous system			
Uneasiness/anxiety	50.5 (47)	50.5 (47)	1
Altered mental status/change in activity level	36.6 (34)	29 (27)	0.274
Loss of consciousness	4.3 (4)	6.5 (6)	0.516

Abbreviation: OFC, oral food challenge.

^a^ *χ* ^2^‐test *p*‐value comparing the proportion of all patients with the specific symptom before versus after the diagnosis was verified by OFC.

^b^ In case of multiple accidental allergic reactions in one patient, the most severe reaction was used for analysis.

^c^ *χ* ^2^‐test *p*‐value comparing Sampson's severity score 1–3 versus 4–5 before versus after the OFC.

In total, 26.1% (93/357) of the patients had experienced at least one accidental allergic reaction before and after the establishment of the food allergy diagnosis by OFC (Table 2). Despite steady proportions of accidental anaphylaxis (72% vs. 78.5%), we found an increase (*p* < 0.001) in the number of patients reporting bronchospasm during accidental allergic reactions from 17.2% before to 43% of patients after the diagnostic OFC. In contrast, cutaneous symptoms such as flushing, generalized urticaria, and angioedema occurred more frequently during accidental allergic reactions that occurred before OFC (*p* < 0.001).

### 3.4 Co‐factors and co‐morbidity in accidental allergic reactions

Co‐factors were rarely implicated in the reported accidental allergic reactions among the patients. Infection within a week (5.6%, 20/357) and physical exercise within 4 hours (5.3%, 19/357) prior to the accidental allergic reaction were the most frequently reported co‐factors in the study population (*n* = 357). On the other hand, mental stress (*n* = 8), alcohol consumption within the same day (*n* = 7), and intake of non‐steroidal anti‐inflammatory drugs (*n* = 1) were rarely reported. Among adults (*n* = 80, ≥18 years), physical exercise (8/80) and alcohol consumption (7/80) were the most common co‐factors. Infections (17/277) and physical exercise (11/277) were the most common co‐factors among children and adolescents (*n* = 277, ≤17 years, data not shown).

Atopic diseases were common co‐morbidities at the time of the most severe accidental allergic reaction. Concomitant asthma diagnosis accounted for 55% (195/357) of the patients, of whom 51.3% (100/195) reported to be well‐treated and 9.7% (19/195) explicitly reported asthma symptoms being uncontrolled. Approximately three‐quarters of the respondents reported current atopic dermatitis (81.2%, 290/357) and allergic rhinoconjunctivitis (73.7%, 263/357).

### 3.5 Treatment of the allergic reactions

In total, 53.8% (192/357) received any treatment for accidental allergic reactions, including oral or intravenous/intramuscular antihistamine or glucocorticoids, inhalation of beta‐2‐agonist or intramuscular adrenaline. However, the proportion increased to 68.3% (144/211) of patients who reported symptoms corresponding to anaphylaxis (Sampson's severity score 3–5; 91/146 aged ≤17 vs. 53/65 aged ≥18), which is a significantly higher treatment proportion compared to patients who reported mild—moderate allergic reactions (Sampson's severity score 1–2, *p* < 0.001). In total, only 68.2% (144/211) of the patients with accidental anaphylaxis (Sampson's severity score of 3–5) responded to the questions regarding treatment with adrenaline auto‐injector. However, among the respondents, 27.1% (39/144) received immediate treatment with an adrenaline auto‐injector, equally distributed (*p* > 0.05) among children/adolescents (age ≤17 years, 25/91) and adults (aged ≥18 years, 14/53).

## 4 DISCUSSION

This survey‐based study characterize accidental food‐allergic reactions outside hospital settings before and after OFC as part of diagnosing food allergy to peanuts, hazelnuts, cow's milk, and hen's egg.

Almost two‐thirds (220/357) of the responders reported accidental food‐allergic reactions that required immediate medical attention, highlighting the importance of implementing preventative measures in clinical practice. However, we observed a decrease in the number of patients experiencing accidental allergic reactions, along with a concurrent increase in the proportion of hospitalizations, when comparing the period after the diagnostic OFC to the period before. Such observations suggest improved disease management by a prophylactic impact from the in‐hospital diagnosis, including OFC, serving as education and potentially improved allergen identification and increased knowledge of threshold for eliciting allergic reactions and recognition of severe allergic symptoms. Furthermore, a particular awareness of respiratory symptoms may explain the increased proportion of accidental allergic reactions elicited after the diagnostic OFC, despite the overall decrease in patients with accidental allergic reactions.

We had no data on which of the up to four potential allergens caused the reported accidental allergic reactions. However, allergies to cow's milk and hen's egg mainly occur in preschool children and are often outgrown before school age, whereas peanuts and tree nut allergies usually appear in later childhood and adolescence^21^ and continue into adulthood.^22^ Given that approximately half of the responding patients were adolescents, it is likely that the time span from their first accidental allergic reaction to the diagnostic OFC was shorter than the period from the OFC to their survey completion. This observation underscores the preventive implications for diagnostic procedures such as OFC in the management of food allergies. However, the role of OFC as part of the diagnostic procedure has been reduced in international guidelines on immunoglobulin E‐mediated food allergy, as the food‐allergy diagnosis may be based on clinical history and sensitization pattern according to in vivo and in vitro tests, whereas OFC remains recommended in unclear cases or to make a definitive diagnosis.^10^ In our allergy centre, most patients suspected of allergy to peanuts, hazelnuts, cow's milk and hen's egg undergo OFC to establish the diagnosis and obtain knowledge of the eliciting threshold for the allergic reactions,^11^ which is essential for tailored guidance.

In this study, 81% of adults reported symptoms during accidental allergic reactions compatible with anaphylaxis, being significantly more frequent among adults than children/adolescents (53%). Such observations are in accordance with recent epidemiological data, suggesting an age‐related increase in severe accidental food‐allergic reactions being common among adolescents and young individuals compared to their pediatric counterparts.^23^ Thus, young adults represent a subpopulation with an increased exhibition of risk‐taking behavior,^24^, ^25^ leading to the highest risk of food‐induced anaphylaxis.^26^, ^27^ Additionally, we found generalized urticaria more frequent in childhood, whereas symptoms from all other organ systems were more frequent in adults. Thus, the clinical manifestations of food‐induced anaphylaxis are heterogeneous and seem to change throughout life, in agreement with previous studies.^28^, ^29^ Thus, the observed decrease in skin symptoms with the concomitant increase in bronchospasm among patients experiencing accidental allergic reactions before and after OFC may be explained by the increased intake of the allergen dose and increased age or may suggest an awareness of respiratory symptoms as a warning sign.^28^ Bronchospasm may also be overestimated among adults when self‐reported, explaining the stationary proportion of accidental food‐induced anaphylactic episodes before and after OFC. Thus, the approach to managing food allergy changes substantially over the life course, emphasizing the potential need for age‐adjusted patient education on accidental allergic reactions. However, we still need data on whether education reduces the risk of severe outcomes,^25^ which is an intuitive presumption.

The lack of correlation between the cumulative threshold for eliciting an allergic reaction during the OFC and the severity of the accidental allergic reactions outside a hospital setting suggests that thresholds do not contribute to the severity of the allergic reactions, in accordance with others.^30^, ^31^ Nonetheless, low thresholds are linked to frequent accidental ingestions and impaired quality of life,^32^ emphasizing the importance of addressing such factors in these patients. Behavioral patterns may account for the lack of association between low cumulative thresholds during OFC and the severity of accidental allergic reactions found in our study. Individuals with low eliciting thresholds are advised to avoid allergens strictly, including reading labels for allergen traces, and carry an AAI. However, a previous study did not find evidence for an association between the OFC threshold and the use of rescue medicine.^33^ Furthermore, real‐life allergic reactions can be exacerbated by various extrinsic non‐allergenic concomitant factors. Such factors were infrequently reported to be implicated in the accidental food‐allergic reactions and were potentially less common than previously reported.^34^, ^35^ Even so, concomitant factors are essential for targeted preventative measures due to their potentially enhancing potential for the development and severity of IgE‐mediated reactions,^36^, ^37^ with uncontrolled asthma remaining a particularly critical intrinsic factor.^38^ However, in retrospective and survey‐based studies, recall bias of co‐factors and co‐morbidities is a substantial risk leading to under‐reporting, as in our study.

Additionally, recall bias may also prompt underreporting of various acute treatment modalities. Administration of AAI was reported among one‐fourth (27%) of patients with food‐induced accidental anaphylactic reaction; however, any treatment for allergic reaction was administered in two‐thirds (68.3%). Thus, despite a higher treatment proportion than other populations,^28^ a significant amount of accidental anaphylactic episodes remained untreated, particularly with first‐line AAI, which emphasizes the importance of patient education on accurate recognition of symptoms and appropriate treatment strategies.^39^

The study possesses strengths in using a comprehensive questionnaire and a large sample size of participants with challenge‐verified food allergies at a tertiary allergy centre. However, limitations include a response rate of 52%, although the respondents were representative of the whole population on essential parameters. Self‐reporting of symptoms during accidental allergic reactions, which potentially dated years prior to questionnaire response, may cause vague estimations of symptom frequencies. Additionally, we lacked data concerning whether peanuts, hazelnuts, cow's milk, or hen's egg elicited individual reactions for the overall estimation of accidental allergic reactions. Given the self‐reported retrospective evaluation of accidental allergic reactions, we had no exact data on the time of the reactions for individual allergens, causing a bias of higher age at the time of questionnaire response and higher number of accidental allergic reactions. Future studies should focus on evaluating the impact of OFC in diagnosing food allergies, including the frequency and severity of accidental allergic reactions, usage of AAI, along with objective data on treatment, co‐factors, and quality of life, optimally evaluated clinically at the time of the accidental food‐allergic reactions.

In conclusion, verifying food allergies through OFC ensures protective measures for the patients, leading to fewer patients with accidental food‐allergic reactions. Children were more likely to experience hives, whereas adults more frequently experienced symptoms from all other organ systems and anaphylaxis. Additionally, co‐factors were infrequently implicated in accidental allergic reactions, and the administration of AAIs was limited.

## AUTHOR CONTRIBUTIONS

**Sebastian Vigand Svendsen**: Writing—original draft; formal analysis; investigation. **Annemarie Schaeffer Senders**: Writing—review and editing; investigation. **Athamaica Ruiz Oropeza**: Writing—review and editing; conceptualization. **Annmarie Lassen**: Writing—review and editing; conceptualization; data curation. **Carsten Bindslev‐Jensen**: Supervision; writing—review and editing; conceptualization. **Charlotte G. Mortz**: Writing—review and editing; investigation; conceptualization; supervision; methodology; project administration; data curation.

## CONFLICT OF INTEREST STATEMENT

The authors declare no conflicts of interest.

## Data Availability

The data are not available due to restrictions from the Regional Committees on Health Research Ethics for Southern Denmark and the Danish Data Protection Agency.
